# Anthropometric Characteristics and Vertical Jump Abilities by Player Position and Performance Level of Junior Female Volleyball Players

**DOI:** 10.3390/ijerph18168377

**Published:** 2021-08-07

**Authors:** Suncica Pocek, Zoran Milosevic, Nemanja Lakicevic, Kristina Pantelic-Babic, Milka Imbronjev, Ewan Thomas, Antonino Bianco, Patrik Drid

**Affiliations:** 1 Faculty of Sport and Physical Education, University of Novi Sad, 21000 Novi Sad, Serbia; suncicapocekfsfv@gmail.com (S.P.); zoranaisns29@gmail.com (Z.M.); milkaimbronjev@gmail.com (M.I.); patrikdrid@gmail.com (P.D.); 2 Sport and Exercise Sciences Research Unit, University of Palermo, 90144 Palermo, Italy; lakinem89@gmail.com (N.L.); antonino.bianco@unipa.it (A.B.); 3 Faculty of Physical Education and Sport, University of Banja Luka, 78000 Banja Luka, Bosnia and Herzegovina; kristina.pantelic-babic@ffvs.unibl.org

**Keywords:** spike jump, block jump, critical threshold, specialization

## Abstract

Although absolute jump heights should be considered an important factor in judging the performance requirements of volleyball players, limited data is available on age-appropriate categories. The purpose of this study is to determine the differences in specific anthropometric characteristics and jumping performance variables in under−19 female volleyball players in relation to playing position and performance level. The sample of subjects consisted of 354 players who prepared for the U19 Women’s Volleyball European Championship 2020 (17.4 ± 0.8 years, 1.81 ± 0.07 m, 67.5 ± 7.1 kg). Playing positions analyzed were setters (*n* = 55), opposites (*n* = 37), middle blockers (*n* = 82), outside hitters (*n* = 137), and liberos (*n* = 43). The results showed player position differences in every performance level group in variables of body height, spike, and block jump. Observed differences are a consequence of highly specific tasks of different positions in the composition of the team. Players of different performance levels are significantly different, with athletes of higher-ranked teams achieving better results. The acquired data could be useful for the selection and profiling of young volleyball players.

## 1. Introduction

Volleyball is a team sport with highly specific tasks and responsibilities for each player on the court according to player’s position [1,2,3]. From the beginners’ level and composition of 6:0, each player goes through a transition period of composition 3:3 and 4:2 to the advanced level of 5:1 team composition, with the highest level of player specialization. Based on anthropometric characteristics, the skill quality and motor abilities of players can be talent-identified and assigned to one of the player’s positions [4,5,6,7,8,9,10,11,12]. The dominant composition played at the highest level of contemporary volleyball is the 5:1 composition. The first number denotes the number of the spikers/hitters, while the second number denotes the number of players who are in charge of the organization of the game in terms of setting. Accordingly, a contemporary volleyball team is composed of 5 hitters, namely, an opposite hitter, two outside hitters, two middle blockers, and the one and only player in charge of the organization of the game—the setter. The “7th” player on the court is a specialist defensive player—the libero—who replaces the middle blocker in serve reception and court defense responsibilities. All of them have highly specific and precise tasks.

Through the long-term process of training, talent identification, and selection, players should distinguish themselves, besides in skill level, in terms of above-average body height, upper and lower muscular power, speed, and agility [13]. Vertical jump is a fundamental part of the spike, block, and serve. At high-level volleyball, jumping is also used while setting because it reduces the flight time of the ball, speeds up the attack, and makes it harder for the first line of defense–block to read through the possibilities of the attacking team. Vertical jump assessment in volleyball is an inevitable part of training and testing procedures [1,14,15,16,17,18,19,20,21]. In a volleyball 5-set match, players in different positions perform a range of jumps that go from 65 to 136 jumps. On average, the highest number of jumps is performed by setters, followed by middle hitters, opposite hitters, and outside hitters [22]. At an elite playing level, a typical match is likely to impose the greatest stress from maximal jumping on middle players, but the setters also perform a very high number of submaximal jumps [1]. 

The purpose of this study is to determine the differences in anthropometric and jumping performance variables in under-19 women volleyball players in relation to playing position and performance level. This information could provide significant insight and reference values for talent identification and evaluation of the training programs applied. 

## 2. Materials and Methods

### 2.1. Sample

The Confederation European de Volleyball U19 Women’s Volleyball European Championship 2020 (CEV U19W ECH 2020) was held in Bosnia & Hercegovina and Croatia from 22–30 August as the first major European competition since the outbreak of the coronavirus (COVID-19) pandemic. Out of the 12 teams initially planned for the championship, Russia, Italy, and Germany withdrew from the competition due to COVID-19 travel restrictions and specific government measures. The subject sample included 354 female volleyball players from the 12 teams planned for the competition and 3 teams that went through the 1st round of the qualification process for the U19 women’s category. 

### 2.2. Data Collection

All data were retrieved from the CEV U19 Women Volleyball webpage (https://www-old.cev.eu/Competition-Area/CompetitionView.aspx?ID=1201) (accessed on 11 September 2020).

Displayed data on the official CEV cite for the abovementioned players included measures of body height, body mass, spike jump, block jump, year of birth, and player position. Body mass index was calculated based on the values of body height and body mass. Team level criteria were composed as follows: Level 1, classified 1st–4th, added by volleyball players from Russia and Italy (CEV U17W ECH 2018 1st and 2nd); Level 2, classified 5th–8th, added by volleyball players from Germany (CEV U17W ECH 2018 7th); and Level 3, which consisted of the 9th team of the final standings of the U19 competition (CEV ranking 17th), added by volleyball players from the 3 teams that played in the 1st round of qualification (CEV ranking 30th−32nd). Player position criteria were observed through the role of setter, opposite hitter, middle blocker, outside hitter, and libero (Table 1). 

### 2.3. Statistical Analysis

Descriptive and inferential analyses of the data were done using the software SPSS v.20 (SPSS Inc., Chicago, IL, USA). Multivariate analysis of variance (MANOVA) with an LSD post hoc test was used to determine the differences in jumping tests and anthropometric measures (dependent variables) between different playing positions and performance levels in volleyball (independent variables). Statistical significance was set at *p* ˂ 0.05.

## 3. Results

Table 2 presents mean (SD) anthropometric, physical, and age characteristics of junior female volleyball players regarding position and performance level. The MANOVAs revealed there are statistically significant differences for player position in 1st (F = 5.69, *p* = 0.00, partial eta-squared = 0.19), 2nd (F = 3.62, *p* = 0.00, partial eta-squared = 0.16), and 3rd performance level groups (F = 3.41, *p* = 0.00, partial eta-squared = 0.22) for body height (1st f = 50.12, *p* = 0.00, partial eta-squared = 0.58, 2nd f = 24.77, *p* = 0.00, partial eta-squared = 0.46, and 3rd f = 26.90, *p* = 0.00, partial eta-squared = 0.59), body mass (1st f = 14.68, *p* = 0.00, partial eta-squared = 0.28, and 2nd f = 8.06, *p* = 0.00, partial eta-squared = 0.22), body mass index (2nd f = 2.71, *p* = 0.03, partial eta-squared = 0.09), spike jump (1st f = 23.99, *p* = 0.00, partial eta-squared = 0.39, 2nd f = 7.70, *p* = 0.00, partial eta-squared = 0.21, and 3rd f = 3.70, p = 0.01, partial eta-squared = 0.17), and block jump (1st f = 15.70, *p* = 0.00, partial eta-squared = 0.30, 2nd f = 6.78, *p* = 0.00, partial eta-squared = 0.19, and 3rd f = 3.21, *p* = 0.02, partial eta-squared = 0.15).

Significant position-related differences in the best performance group in terms of body height are evident in all positions except between opposite and middle blocker players (post hoc LSD *p* = 0.54), with greater values of both opposites and middles in comparison with outside hitters, after whom are setters and libero players. For spike and block jumps, there are no significant differences between players in the positions of opposite and middle blocker (post hoc LSD for spike *p* = 0.24 and block 0.06) as well as the middle blocker and outside hitter (post hoc LSD for spike *p* = 0.11 and block 0.42), while there are statistically significant differences between opposites and outside hitters (post hoc LSD for spike *p* = 0.02 and block 0.01), with better results for opposite players. In all other mutual relations, there are statistically significant differences in the following order: from opposites, middles, outside hitters, setters and libero players, from best to worst results in spike and block jump. In the 2nd performance group, there are no significant differences between opposites and outside hitters in body height, spike, and block jump (post hoc LSD for body height *p* = 0.72, spike 0.28, and block 0.32). In the lowest performance group (3rd) of young volleyball players, differences in the abovementioned variables between positions are even less pronounced significant differences are not observed, although it was expected otherwise (for example, between setters and opposites, post hoc LSD for spike *p* = 0.97 and block 0.40).

The MANOVAs revealed there are statistically significant differences by performance level in the player position of opposites (F = 2.23, *p* = 0.02, partial eta-squared = 0.31), middle blockers (F = 2.87, *p* = 0.00, partial eta-squared = 0.19), outside hitters (F = 5.77, *p* = 0.00, partial eta-squared = 0.21), and libero players (F = 2.03, *p* = 0.03, partial eta-squared = 0.25). Univariate analysis showed that there are statistically significant differences in the observed variables: for opposites, in body height (f = 7.52, *p* = 0.00, partial eta-squared = 0.31), spike jump (f = 12.04, *p* = 0.00, partial eta-squared = 0.41), and block jump f = 12.22, *p* = 0.00, partial eta-squared = 0.42); for middle blockers, in body height (f = 6.13, *p* = 0.00, partial eta-squared = 0.13), body mass (f = 4.49, *p* = 0.01, partial eta-squared = 0.10), spike jump (f = 17.90, *p* = 0.00, partial eta-squared = 0.31), and block jump (f = 5.39, *p* = 0.01, partial eta-squared = 0.12); for outside hitters, in body height (f = 23.67, *p* = 0.00, partial eta-squared = 0.26), body mass (f = 13.19, *p* = 0.00, partial eta-squared = 0.17), body mass index (f = 8.93, *p* = 0.00, partial eta-squared = 0.12), spike jump (f = 10.96, *p* = 0.00, partial eta-squared = 0.14), and block jump (f = 7.65, *p* = 0.00, partial eta-squared = 0.10). Based on the post hoc LSD test, we can observe that the 1st and 2nd group of opposites, middles, outsides, and liberos, in comparison to the 3rd group, have greater values of body height and better results of spike and block jump. In the player position of opposites, there are also statistically significant differences between 1st and 2nd performance levels. Differences in the varied performance levels of setters with greater values of body height and better results of spike and block jump of the 1st performance level in comparison to 2nd and 2nd in comparison to 3rd are observed, although they are not statistically significant. 

Finally, Table 3 reports position-specific normative centile values for anthropometric characteristics in terms of body height and sport-specific jumping abilities in absolute values, i.e., spike jump and block jump. 

## 4. Discussion

The aim of this study is to investigate the anthropometric characteristics and vertical jumping abilities of junior female volleyball players according to player position and performance level. The main results of our study are as follows: 

(a) there are statistically significant differences by player position in every performance level group in the variables of body height, spike jump, and block jump. 

(b) Significant position-related differences in the best performance group in terms of body height are evident in all positions except between opposite and middle blocker players, with greater values of both opposites and middles in comparison to outside hitters, followed, in order, by setters and libero players. In the variables of spike and block jumps, there are no significant differences between players in the positions of opposite and middle blocker, as well as middle blocker and outside hitters, while there are statistically significant differences between opposites and outside hitters, with better results from opposite players. In all other mutual relations, there are statistically significant differences in the following order: from opposites, middles, outside hitters, setters till the libero players, and from best to worst results in spike and block jumps. In the 2nd performance group, the same conclusions were derived with the addition that in this group, there were no significant differences between opposites and outside hitters in body height, spike jump, and block jump as a consequence of the lower values of opposites of the 2nd performance level group in comparison with the 1st performance level group, which were leveled to the values of the outside hitters. In the lowest performance group (3rd) of young volleyball players, differences in the abovementioned variables between positions were even less pronounced and non-significant. 

(c) There are statistically significant differences by performance level, with greater values of body height and better results of spike and block jumps of the 1st and 2nd group of opposites, middles, outsides, and liberos in comparison to the 3rd group. In the player position of opposites, there are also statistically significant differences between 1st and 2nd performance levels. Differences in the varied performance levels of setters with greater values of body height and better results of spike and block jumps of the 1st performance level in comparison to 2nd and 2nd in comparison to 3rd are observed, although they are not statistically significant. 

Based on the results of the present research, the data showed that there are statistically significant differences in body height and absolute values of spike and block jumps between positions in volleyball. These findings are in accordance with research conducted by different authors [1,3,23], and they are within expectations due to player tasks on the court. At the same time, numerous studies that had taken into account the relative values of jumping abilities did not find any differences between player positions except for body height values [2,24,25,26]. Consequently, we can see on the basis of the norms of absolute values (Table 3), which is the critical height that athletes should reach. Elite volleyball players need to reach the threshold in the absolute values of spike and block jumps for specific positions. Such can be achieved either on the count of above-average body height and/or relative values of vertical jump in order to reach that threshold. Those players with a lower body height can compensate for their lack by an above-average jumping ability for the particular position that is targeted. In Table 3, we can see to which extent it should be expected. In such a manner, differences in relative jumping abilities between player positions are possible [19]. 

In this respect, relative vertical jumping ability is of great importance in volleyball regardless of the players’ position, while absolute vertical jump values can differentiate players not only in terms of player position and performance level but in their career trajectories. However, maximum jump height performance in each and every jump, either in the spike or in the block, is neither necessary nor expedient. Due to player adaptation of their efforts to the game situation and efficacy of their performance throughout the game, the intensities of attack jumps at maximal capacity varies from 55–90% [27]. A higher contact height in the attack motion involves a better incidence angle of the opponent court [28]. Differences between the values of spike and block jumps, with the greater reach of spike jumps, are due to the type of the approach (frontal vs. lateral) and how the ball is contacted (one hand vs. both hands simultaneously). The fact that the aim of block performance is to increase the area by which we limit attacker options, the player needs to place both hands simultaneously on the ball when performing the block. Additionally, the reason for such height discrepancies between spike and block jumps is that the first is executed individually while a block must be performed by two or three players in a coordinated manner in which players need to adjust and harmonize their temporal and spatial actions. 

Player specialization, i.e., determining the player’s position, is a complex and long-term process. Based on the player’s characteristics and abilities, coaches should assign the player a role on the court that would maximize the player’s contribution to the team. Coaches may sometimes encounter resistance from players due to their affinities, but the specialization process should be approached thoroughly. Talent identification and development is a process based on an understanding of the tasks and responsibilities of the player regarding their position (Table 4) as well as a consideration of the body measures and abilities associated with sports performance. In pursuit of elite performance, it is of great importance to differentiate between the trainable and non-trainable qualities of a player [29,30,31].

A spike is the most attractive and efficient way of scoring a point. The success of this action depends on height of contact, ball direction, and ball speed. The main factors that determine the height of contact are standing height reached, which consists of body height and arm length, and ability to jump and reach, which consists of the ability of a player to perform technical elements in the most efficient way in terms of the utilization of motor abilities, namely, explosive muscular power. For the height of contact, the ability to jump and reach is usually monitored in volleyball [15]. With the exception of liberos, every player may spike. 

Throughout the years, as demonstrated in the European and World Championships, there has been an increase in the use of the power jump serve in both men’s and women’s volleyball [32]. With the change in rules and the introduction of the Rally Point System, the serve as a skill has become a mighty tool for scoring a point and not just for entering the rally in the competition of two teams. Even when a team does not score a direct or ace point, through a powerful power jump serve, it can reduce the possibilities of attack from the opponent team and facilitate the organization of a counterattack. Therefore, while performing spikes, power serves (which is very similar to the spike technique), and blocks, volleyball players should possess above average body height in combination with the power and force of lower extremities in executing simple vertical jumps. 

Because of the similar requirements in spike and block jumps for opposite (main hitter) and middle blockers (main blocker), the shorter outside hitters, in order to reach the critical threshold, must make up for their lack of height and standing reach by exhibiting superior relative jump heights. In this respect, absolute jump heights of spike and block are of great importance when judging the performance requirements of outsides. Those players who did not reach these thresholds cannot play at the elite level of volleyball on the position of outsides. Hence, these players were left out during the transition from junior to senior levels of competition, which resulted in their playing career coming to an end. Because of their exquisite skillfulness in serve reception and court defense, in order to keep them in the team and improve the game, in 1998, there was a change in rules and the introduction of the libero player. 

Our study was able to identify differences between various playing positions in terms of body height and absolute vertical jump for both spike and block in elite junior female volleyball players. However, the main limitation of this study is that all data were retrieved from the data displayed by the official CEV site from the competition of the U19 Women’s Volleyball European Championship 2020. 

## 5. Conclusions

Player specialization, i.e., determining the player’s position, is a complex and long-term process. Based on the player’s characteristics and abilities, coaches should assign the player a role on the court that would maximize the player’s contribution to the overall quality of the team. During that process, it is important to differentiate between the trainable and non-trainable qualities of a player.

Based on the results of this research, the data shows that there are statistically significant differences in body height and absolute values of spike and block jumps between positions in volleyball. Relative vertical jumping ability is of great importance in volleyball regardless of the players’ position, while absolute vertical jump values have the power to differentiate players not only in terms of player position but also in performance level. The higher the performance level of the team, the lower the intra-positional differences in terms of height, spike jump, and block jump, and some other factors become decisive (e.g., technical–tactical skill and knowledge, decision-making quality, performance under pressure).

Deficit of body height for a particular position can be compensated by jumping ability only to some extent. The relatively large sample of subjects in our study is composed of elite (1st group), good (2nd group), and lower levels of performance (3rd group) of U19 women volleyball players. In pursuit of excellence and competition on the elite senior level, they need to reach the threshold for their age and particular position in terms of absolute spike and block jumps based on the normative values presented. 

## Figures and Tables

**Table 1:** Number of players in each position and each team, analyzed according to the CEV U19 Volleyball European Championship 2020 data.

Performance Level	Setter	Opposite	Middle Blocker	Outside	Libero	∑
1. Turkey	5	2	7	15	3	32
2. Serbia	4	2	5	15	3	29
3. Belarus	3	2	5	5	3	18
4. France	4	0	8	8	2	22
* Russia	2	4	6	7	5	24
* Italy	3	5	6	11	4	29
∑ level 1	21	15	37	61	20	154
5. Poland	4	1	6	8	3	22
6. Bulgaria	5	4	6	13	2	30
7. Croatia	3	3	4	8	4	22
8. Slovakia	3	2	6	7	4	22
* Germany	4	2	6	10	3	25
∑ level 2	19	12	28	46	16	121
9. Bosnia and Herzegovina	5	4	5	8	2	24
^§^ Sweden	3	1	3	8	2	17
^§^ Israel	3	1	6	6	2	18
^§^ Montenegro	4	4	3	8	1	20
∑ level 3	15	10	17	30	7	79
Total	55	37	82	137	43	354

* Teams which did non participate in the competition due to COVID restritions. ^§^ Teams which played only the 1st round of qualification.

**Table 2:** Anthropometric, physical, and age characteristics of junior female volleyball players regarding position and performance level.

	Performance Level	Setter ^1^	Opposite ^2^	Middle Blocker ^3^	Outside ^4^	Libero ^5^	∑
Body height (m)	1	1.81 ± 0.06 ^5^	1.88 ± 0.03 ^1,4,5^	1.87 ± 0.04 ^1,4,5^	1.84 ± 0.04 ^1,5^	1.71 ± 0.05	* 1.83 ± 0.07
	2	1.78 ± 0.06 ^5^	1.84 ± 0.04 ^1,5^	1.87 ± 0.05 ^1,3,5^	1.83 ± 0.05 ^1,5^	1.73 ± 0.04	* 1.82 ± 0.07
	3	1.77 ± 0.03 ^5^	1.81 ± 0.06	1.83 ± 0.04 ^1,4,5^	1.78 ± 0.04 ^5^	1.62 ± 0.05	* 1.78 ± 0.07
	∑	1.79 ± 0.05	^‡^ 1.85 ± 0.05	^‡^ 1.86 ± 0.05	^‡^ 1.82 ± 0.05	^‡^ 1.71 ± 0.06	1.81 ± 0.07
Body mass (kg)	1	68.4 ± 7.0 ^5^	72.1 ± 5.5 ^5^	73.3 ± 5.9 ^4,5^	70.3 ± 5.6 ^5^	61.5 ± 5.1	* 69.8 ± 6.8
	2	63.8 ± 5.0	66.6 ± 7.1 ^5^	71.3 ± 7.0 ^1,2,4,5^	65.8 ± 5.5 ^5^	62.1 ± 4.3	* 66.3 ± 6.5
	3	65.2 ± 6.9	65.9 ± 10.9	67.8 ± 6.3	64.6 ± 6.3	58.3 ± 5.6	65.0 ± 7.3
	∑	66.0 ± 6.5	68.6 ± 8.1	^‡^ 71.5 ± 6.6	^‡^ 67.5 ± 6.2	61.2 ± 5.1	67.5 ± 7.1
BMI (kg/m^−2^)	1	21.0 ± 2.0	20.5 ± 1.3	21.0 ± 1.4	20.8 ± 1.6	20.9 ± 1.3	20.8 ± 1.6
	2	20.1 ± 1.4	19.7 ± 1.8	20.3 ± 1.6 ^4^	19.5 ± 1.2	20.7 ± 1.5^4^	* 20.0 ± 1.5
	3	20.7 ± 1.8	20.1 ± 2.3	20.3 ± 1.6	20.4 ± 1.8	22.1 ± 1.8	20.6 ± 1.8
	∑	20.6 ± 1.8	20.1 ± 1.7	20.6 ± 1.6	^‡^20.3 ± 1.6	21.0 ± 1.5	20.5 ± 1.6
Spike jump (m)	1	2.89 ± 0.09 ^5^	3.04 ± 0.12 ^1,4,5^	3.00 ± 0.09 ^1,5^	2.97 ± 0.10 ^1,5^	2.77 ± 0.11	* 2.95 ± 0.13
	2	2.87 ± 0.10 ^5^	2.90 ± 0.10 ^5^	2.95 ± 0.10 ^1,5^	2.94 ± 0.12 ^1,5^	2.78 ± 0.16	* 2.91 ± 0.13
	3	2.75 ± 0.16 ^5^	2.75 ± 0.21 ^5^	2.81 ± 0.16 ^5^	2.84 ± 0.16 ^5^	2.59 ± 0.15	* 2.78 ± 0.18
	∑	2.84 ± 0.13	^‡^ 2.92 ± 0.19	^‡^ 2.95 ± 0.13	^‡^ 2.93 ± 0.13	^‡^ 2.74 ± 0.15	2.90 ± 0.15
Block jump (m)	1	2.75 ± 0.09 ^5^	2.92 ± 0.11 ^1,4,5^	2.85 ± 0.16 ^1,5^	2.83 ± 0.10 ^1,5^	2.63 ± 0.14	* 2.80 ± 0.15
	2	2.76 ± 0.10 ^5^	2.77 ± 0.10 ^5^	2.83 ± 0.10 ^1,5^	2.80 ± 0.11 ^5^	2.65 ± 0.16	* 2.78 ± 0.13
	3	2.65 ± 0.18 ^5^	2.58 ± 0.28	2.70 ± 0.20 ^5^	2.71 ± 0.18 ^5^	2.44 ± 0.21	* 2.66 ± 0.21
	∑	2.73 ± 0.13	^‡^ 2.78 ± 0.22	^‡^ 2.81 ± 0.16	^‡^ 2.79 ± 0.13	^‡^ 2.61 ± 0.17	2.76 ± 0.17
Age (years)	1	17.7 ± 0.6	17.0 ± 1.0	17.5 ± 0.8	17.3 ± 0.9	17.6 ± 0.7	17.4 ± 0.8
	2	17.6 ± 0.5	17.2 ± 0.8	17.4 ± 0.6	17.3 ± 0.9	17.4 ± 0.7	17.4 ± 0.8
	3	17.5 ± 0.9	16.9 ± 1.1	17.4 ± 0.7	17.2 ± 0.9	17.4 ± 0.8	17.3 ± 0.9
	∑	17.6 ± 0.7	17.0 ± 1.0	17.5 ± 0.7	17.3 ± 0.9	17.5 ± 0.7	17.4 ± 0.8

Significantly different from: ^1^—Setter; ^2^—Opposite; ^3^—Middle blocker; ^4^—Outside; ^5^—Libero; *—significantly different by player position; ‡—significantly different by performance level.

**Table 3:** Position-specific normative centile values of body height, spike jump, and block jump for junior female volleyball players.

		Setter			Opposite			Middle Blocker			Outside			Libero		
		Bh	SJ	BJ	Bh	SJ	BJ	Bh	SJ	BJ	Bh	SJ	BJ	Bh	SJ	BJ
Percentile cut-off	5	1.71	2.53	2.45	1.73	2.49	2.20	1.78	2.71	2.57	1.74	2.73	2.60	1.58	2.45	2.32
	10	1.73	2.71	2.58	1.76	2.63	2.47	1.80	2.79	2.68	1.76	2.79	2.67	1.64	2.53	2.40
	25	1.75	2.78	2.66	1.82	2.83	2.69	1.82	2.88	2.75	1.80	2.85	2.71	1.66	2.66	2.50
	50	1.79	2.85	2.75	1.85	2.94	2.83	1.86	2.97	2.82	1.82	2.95	2.80	1.70	2.74	2.60
	75	1.81	2.93	2.80	1.88	3.03	2.90	1.89	3.04	2.91	1.85	3.01	2.89	1.75	2.85	2.76
	90	1.86	3.00	2.88	1.91	3.09	3.00	1.92	3.09	2.98	1.88	3.09	2.95	1.78	2.95	2.82
	95	1.91	3.01	2.90	1.92	3.19	3.03	1.95	3.11	3.00	1.90	3.15	3.00	1.80	2.98	2.85

Values of body height, spike jump, and block jump are presented in meters.

**Table 4:** Player’s role and subsequent tasks in volleyball according to their position.

	Setter	Opposite	Middle	Outside	Libero
Serve	✓	✓	✓	✓	-
Serve reception	-	-	-	✓	✓
Setting	✓ (II–III)	-	- (^4^)	-	- (^4^)
Spike	(^1^ IV, III, II)	II, I (^2^ R1 IV)	III	IV, VI (^2^ R1 II)	-
Block	II	II (^3^ IV)	(IV, III, II)	IV (^3^ II)	-
Backcourt defense	I	I	V	VI	V

✓: Performed task by role, roman letters indicate the field zone in which such task can be performed. ^1^ Setters do not perform spikes, but are allowed in certain situations: setter positioned in Zone IV, Zone III, and Zone II. ^2^ R1, setter in Zone I: opposite player performs spike from Zone IV, while outside hitter performs spike from Zone II. ^3^ R1, setter in Zone I: as a response to an opponent’s counterattack, the opposite player performs a block from Zone IV while the outside hitter performs a block from Zone II. ^4^ if setter is not able to perform setting due to previous contact with the ball, middle blocker or libero could help.

## Data Availability

The data presented in this study are available on request from the corresponding author.
